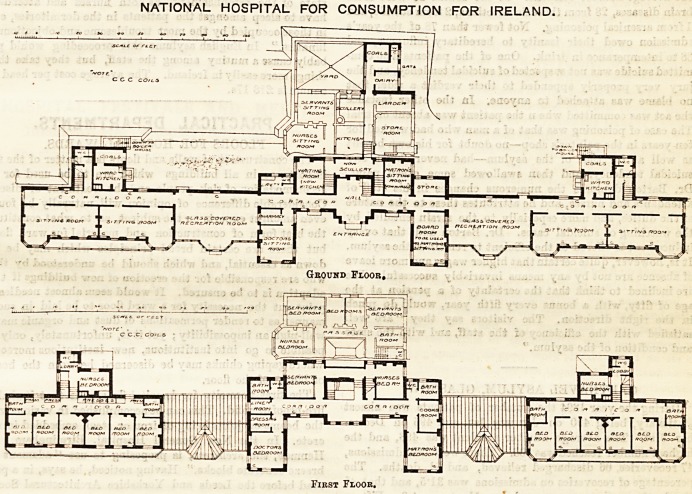# Hospital Construction

**Published:** 1895-06-08

**Authors:** 


### THE INSTITUTIONAL WORKSHOP.

### HOSPITAL CONSTRUCTION.

### NATIONAL HOSPITAL FOR CONSUMPTION FOR IRELAND.

This hospital is in course of erection at Newtown, Mount Kennedy, co. Galway. The site covers an area of nearly twenty acres, occupying the southern slope of an eminence that rises about 300 feet above sea-level, and commands extensive views of rich and undulating scenery of great beauty. The buildings are so arranged that all the patients' rooms face south, and while freely exposed to the sun are well sheltered from the north and east winds. The centre or administration building contains the rooms for the resident physician, and those for the matron, together with the kitchen offices, and bed-rooms for nurses and servants. This block it is intended at some future time to increase by building out at the back. Meanwhile the matron's sitting-room is used by the nurses, the matron herself using the board-room as her sitting-room, the nurses' sitting-room being now used as kitchen.

On each side of the centre block is a large glass-roofed recreation-room. These give access on either side to the wings which contain the patients' accommodation.

The patients' blocks are three storeys in height, and afford accommodation for twelve patients in each. On the ground floor on each side are two very spacious sitting-rooms, each provided with a bay window, and having a verandah in front. At the back is a ward kitchen, with a w.c., lavatory, and coal store.

On the upper floors are the patients' bed-rooms, each patient having a separate room, provided with a fire-place, and, as before stated, facing south. On each floor at each end of the corridor is a bath-room, and in the centre a nurse's bed-room, the staircase, and a w.c., with a lobby interposed between it and the passage. The patients' bed-rooms all have access to a verandah.

The plans also provide for the addition, when funds will permit, of a large central dining hall.

The system of heating and ventilation will, we understand, provide for a constant supply of 5,000 cubic feet of fresh air per patient per hour warmed in the coldest weather to a temperature of 50° Fahr., and an equal amount of vitiated air is to be extracted by mechanical means. We have no information on the particular means to be adopted to obtain this result, and can, therefore, express no opinion on the matter. A bountiful supply of fresh air is of immense value to sufferers from acute lung disease is beyond question, while at the same time it is of the utmost importance to preserve an equable and sufficiently high temperature and to avoid draughts.

The buildings have been conceived in no niggardly spirit, and the general arrangements appear to have been carefully thought out in every particular. The architects are Sir Thomas N. Deane and Son, of Dublin.

NATIONAL HOSPITAL FOR CONSUMPTION FOR IRELAND.

GROUND FLOOR.

FIRST FLOOR.

## Figures and Tables

**Figure f1:**